# Glucose Metabolism in Turner Syndrome

**DOI:** 10.3389/fendo.2019.00049

**Published:** 2019-02-07

**Authors:** Lin Sun, Yao Wang, Tong Zhou, Xue Zhao, Yingxuan Wang, Guixia Wang, Xiaokun Gang

**Affiliations:** ^1^Department of Endocrinology, First Hospital of Jilin University, Changchun, China; ^2^Department of Orthopedics, The Second Hospital Jilin University, Changchun, China

**Keywords:** turner syndrome, glucose metabolism, insulin resistance, diabetes mellitus, growth hormone, estrogen

## Abstract

Turner syndrome (TS) is one of the most common female chromosomal disorders. The condition is caused by complete or partial loss of a single X chromosome. Adult patients with TS have a high prevalence of diabetes mellitus (DM). Deranged glucose metabolism in this population seems to be genetically triggered. The traditional risk factors for DM in the general population may not play a major role in the pathogenesis of DM in patients with TS. This review focuses on the latest research studies pertaining to abnormalities of glucose metabolism in TS. We extensively review the available evidence pertaining to the influence of insulin secretion and sensitivity, obesity, autoimmunity, lifestyle, growth hormone, and sex hormone replacement therapy on the occurrence of DM in these patients.

## Introduction

Turner syndrome (TS) is one of the most common chromosomal disorders of female development. The estimated prevalence of TS is 25–50 per 1,00,000 females (1). The condition is caused by complete or partial deletion of an X chromosome in all or some of the somatic cells (2, 3). The diagnosis is based on the karyotype analysis of peripheral blood lymphocytes (4). About 50% of patients have haplotype 45, X, while about 20–30% have chimerism 45, X/46, XX, 45, X/47, XXX, and some chromosomal structural abnormalities. The typical symptoms of TS include short height, webbed neck, low hairline at the back of the neck, low-set ears, markedly elevated levels of follicle stimulating hormone (FSH), chronic otitis media (OM), lymphedema of extremities, small mandible, and multiple pigmented nevi (4). Patients with TS are often affected by many other comorbidities, including autoimmune diseases (AD), hypothyroidism, kidney dysfunction, loss of ovarian function or other reproductive disorders, neurological or ophthalmological abnormalities, osteoporosis, diabetes mellitus (DM), dyslipidemia, hypertension, and heart disease (2, 5–10).

The increased incidence of DM in TS patients was first reported almost 50 years ago by Ann Forbes and Eric Engel (11). Abnormal glucose metabolism is found in >70% of adults affected by TS (12, 13); the abnormalities include impaired glucose tolerance (IGT), hyperinsulinaemia, and reduced insulin sensitivity. Increased prevalence of DM coupled with higher incidence of cardiovascular disease in patients with TS may contribute to increased mortality in this population. The specific DM phenotype associated with TS remains unclear. An epidemiologic study in Denmark showed that the incidence of type 1 diabetes mellitus (T1DM) and type 2 diabetes mellitus (T2DM) in TS patients is 11 times and 3–4 times greater than that in healthy people, respectively (14). However, clinical endocrinal studies in adult populations suggest that the phenotype of gradually progressive, adult-onset glucose intolerance is more likely to be T2DM (13, 15). Indeed, the link between TS and T1DM is not well characterized. In the National Cooperative Growth study in the United States, the incidence of T1DM among girls with TS (*n* = 5,220) was greater than that in the general population; however, the standardized incidence ratio (SIR) was not statistically significant (SIR, 0.92–4.18) (16). According to the latest clinical practice guidelines for TS, the prevalence of glucose intolerance and T2DM in patients with TS is 15–50 and 10%, respectively; however, the prevalence of T1DM is yet to be determined (2). It is hypothesized that occurrence of DM in TS is linked to insulin resistance (15) or impaired β-cell function (17). However, the precise mechanism of development of DM in this patient population is not clear.

This review focuses on the latest updates about the pathogenesis of deranged glucose metabolism in TS and the effect of routine TS therapy on the glucose metabolism in these patients. Beginning with current controversies related to the mechanisms, we review the latest evidence pertaining to the intrinsic risk factors, potential confounding variables, and the effect of growth hormone (GH) and EP (Estrogen/Progestin) therapies on DM incidence. The objective of this review is to provide insights into the treatable and non-treatable risk factors for development of DM in TS patients.

## Overview of Glucose Metabolism in TS Patients

Recent work has highlighted that abnormal glucose metabolism is common in patients with TS. Currently, most researchers tend to use oral glucose tolerance test (OGTT) to study glucose metabolism in TS patients. Studies have shown that OGTT is superior to other tests (fasting blood glucose, postprandial blood glucose, or glycosylated glycoprotein levels) for diagnosis of early abnormalities of glucose metabolism in these patients (18).

The incidence of IGT in TS patients is about 10–34%, which is higher than that in the healthy population. Moreover, even though some patients with TS have normal fasting blood glucose and glycated hemoglobin levels, the incidence of IGT is generally higher than that in the healthy subjects, irrespective of the age of the patient (TS girls or TS adult women) (19). In order to rule out the negative effects of obesity and gonadal dysplasia on glucose metabolism, Bakalov et al. compared TS patients with age- and body mass index (BMI)-matched control group with normal karyotype but premature ovarian failure (17). The incidence of IGT in TS group was still significantly higher than that in the control group.

Some studies have shown a stronger correlation of age with the occurrence of abnormal glucose metabolism in TS patients. In a study by Cicognani et al. (20), the incidence of IGT in TS children (age: 5–12 years) was 40%, while the incidence in TS adolescents (age: 12–16 years) was 23.5%, however, the corresponding incidence in adult women was 25–78% (7, 12, 17). In a recent cohort study of 103 patients with TS by Lebenthal et al. (1), the proportion of patients with elevated fasting blood glucose level was 6.6%, while the corresponding proportion among children and adolescents was 8.1%. In contrast, the proportion of patients with IGT was found to increase with increase in age (children, 10%; adolescents, 16.7%; young adults, 21.4%; and adults, 41.2%). Similar findings were reported by Ibarra-Gasparini et al. (13); they also found no association of the traditional risk factors for T2DM (BMI, body composition, family history) or history of growth hormone or sex hormone replacement therapy with impaired glucose tolerance. In addition, age was the only independent predictor of DM in patients with TS.

### Genetic Mechanisms of Impaired Glucose Metabolism in TS Patients

It seems that the disordered glucose metabolism in TS patients is caused by the characteristic changes associated with the disease itself. It has been hypothesized that this may result from deletion of some genes related to insulin signal transduction and β cell function located on the X chromosome.

Bakalove et al. (12) explored the possible mechanisms at the gene level; the incidence of T2DM in patients with TS was 25% (56/224), while only 1 patient had T1DM. After karyotyping, patients with 45, X and X short arm deletion (delXp) had a relatively high incidence of DM (17 and 23%, respectively), while those with X chromosome long arm loss (delXq) had a lower incidence of DM (9%). The results showed that the Xp chromosome haploid gene deficiency increases the risk of DM in the TS population.

The major X-chromosome pseudoautosomal region (PAR1) is located at the Xp end, and the lack of expression of the haploid gene is thought to be related to certain phenotypes of TS. For example, the *SHOX* gene plays a critical role in bone growth and development and its deficiency is liable to cause short height of patients with TS. *PAR1* genes (21) encode several types of receptors, phospholipases, protein phosphatases, GTP binding proteins, ATP transporter, and transcription factors. Therefore, PAR1 haploid gene deficiency may affect the insulin response by affecting the expression of the above molecules. Studies have also shown that the long arm of the X chromosome (iXq) is associated with a higher incidence of DM (43%) as compared to the 45 and X groups. Therefore, it is speculated that additional Xq copies may trigger escape-inactivated gene overexpression, including diabetes-related genes such as islet cell antigen (*ICA*), C-reactive protein (*CRP*), insulin-like growth factor-II (*IGFI-II*), and other genes related to the normal physiological functions of islet cells (*GLIS3, KLF11*) (12). This hypothesis was confirmed by comparing the relative gene expressions of 45, X (*n* = 10) with 46, X, i (X) (q) (*n* = 5) TS patients. Some researchers have found that genes encoding GAD and ICA overexpression are closely related to immunologic injury of β-cells. Therefore, it was speculated that high incidence of DM in the 46, X, i (X) (q) group was linked to the production of β-cell autoantigen. Supernumerary copies of Xq may increase the risk of diabetes even in those who do not exhibit monosomy X, e.g., among men with Klinefelter syndrome (47, XXY) and 48, XXYY (22).

In summary, it is currently believed that the high incidence of DM in the TS population may be due to Xp haplotype gene deficiency, which leads to impaired β-cell function; in addition, overexpression of some genes of Xq may aggravate the problem.

### Insulin Sensitivity in TS Patients

Studies pertaining to insulin sensitivity in patients with TS have yielded inconsistent results. Most studies suggest that TS patients have impaired insulin sensitivity. Choi et al. evaluated insulin sensitivity using the insulin sensitivity quantification index (QUICKI) in two groups of patients with impaired glucose tolerance and normal glucose tolerance; they found reduced insulin sensitivity in the IGT group. In addition, Mazzanti et al. (19) investigated the use of growth hormone therapy and insulin sensitivity in TS patients; the results showed that patients with TS have mild insulin resistance even prior to initiation of growth hormone (GH) replacement therapy. Decreased insulin sensitivity in TS patients was also uncovered in a study that compared TS patients with age- and BMI-matched controls with premature ovarian failure (17). Salgin et al. (15) also provided evidence of insulin resistance in TS patients; they suggested that insulin resistance in TS patients is not a consequence of changes in body fat or body mass. However, other studies that employed the same methods for assessment of insulin sensitivity assessment showed normal insulin sensitivity in TS patients. For example, both Hjerrild et al. (23) and Bakalov et al. (17) (QUICK study) used insulin clamp technique and found that the insulin sensitivity of TS patients is similar to that in the age- and BMI-matched normal control group.

Women with TS who have X-monosomy and X mosaicism were found to develop higher levels of insulin resistance at a younger age relative to that in age-matched controls (20). Insulin receptor substrate 4 is encoded in the Xp22.3-23 region, and it may be linked to insulin resistance in other contexts (15, 24). More work is required to identify the specific genes on the X chromosome that contribute to impaired insulin sensitivity.

### Insulin Secretion in TS Patients

Several studies have demonstrated that abnormal insulin secretion is the main mechanism of development of DM in TS patients. In a cross sectional study of TS patients by Hjerrild et al. (23), insufficient compensatory insulin production during GTT was shown to result in an overall reduction in the insulin-to-glucose ratio; in addition, there was evidence of declining β-cell function. Ibarra-Gasparini et al. (13) conducted a clinical study of 113 patients with TS. During OGTT in TS patients with DM, there was insufficient insulin secretion in the first phase (60 min) after the glucose load; however, in the subsequent 60 min, there was a certain increase in insulin levels. A recent study further indicated that in TS women, traditional factors underlying insulin resistance may not be the reason of their phenotype; instead, deficiency of pancreatic beta cells may lead to impaired glucose tolerance, thereby leading to development of DM over a period of time (9). It appears that decreased insulin secretory response may be the underlying mechanism of the observed increase in the risk of DM in TSpatients.

## Obesity and Body Composition in TS Patients

Obesity, increased waist circumference, and high adipose content may further contribute to the increased prevalence of DM in TS patients. Patients with TS have a high prevalence of obesity owing to the impaired fitness and typically sedentary life as a result of the syndrome (25). The average waist circumference of these individuals is larger, consistent with an increase in abdominal fat, which is a risk factor for T2DM as well (9). Higher BMI values during childhood strongly predict the occurrence of obesity at later ages (26). In a large retrospective cohort study of TS patients, patients who developed obesity and cardiovascular diseases in early childhood and who showed an increasing tendency with age, were more likely to suffer from metabolic disorders such as T2DM during adolescence than non-obese healthy people (1).

Nonetheless, whether BMI, adiposity, and waist circumference are truly correlated with T2DM in patients with TS as they do in the general population remains controversial. Hamilton et al. (9) suggested that while BMI can be a good measure of fat tissue levels in a large population, in a smaller subgroup, the BMI standard deviation score (BMI-SDS) offers superior insights as it is better controlled for age in such settings. Interestingly, BMI-SDS scores of patients with TS and matched controls were found to be comparable, and this finding was further supported by an MRI assessment of adipose tissue levels. In the same study, some of the TS patients that presented cardiovascular and metabolic problems in childhood exhibited normal BMI, which further raises doubts on the link between BMI and T2DM in this population (1). Indeed, age and specific karyotype seem to be more predictive of abnormal glucose metabolism in patients with TS (20). TS may itself increase metabolic abnormalities during childhood, which consequently increases the prevalence of obesity among TS patients (1). Obesity may in turn further disrupt normal glucose metabolism and induce insulin resistance in a feedback loop, which ultimately accelerates the development of T2DM in patients with TS.

The average height of TS patients is 20 cm lower than that of their age-matched peers; the altered growth is restricted to height and does not affect the horizontal growth (27). As a consequence, the arm span and sitting height are roughly 3 standard deviations lower than that of the general population, while head circumference and hand/foot size are typically comparable. Therefore, the total and visceral fat mass in TS patients is generally elevated, and lean body mass (LBM) as well as skeletal muscle mass are decreased. General physical activity and VO2 max (maximal oxygen consumption) are also substantially decreased in those with TS (7). Similarly, fat mass in the arms, legs, and the torso of TS patients is higher than that in controls (28); this may potentially promote insulin resistance and T2DM in these patients. Gravholt et al. (7) also identified enlargement of type IIa muscle fibers in TS patients, which was related with lower requirement of oxygen and glucose content for normal metabolic activity.

Adipose tissue is currently considered as an endocrine tissue, which actively produces a wide range of bioactive adipokines including adiponectin, chemerin, and vaspin. These adipokines can regulate fat mass and the activity of adipocytes. These chemokines can broadly influence the cardiovascular and neuroendocrine systems, leading to altered glucose and lipid metabolism (29). Due to the broad regulatory roles, adipokine dysregulation in patients with TS may further contribute to the abnormal metabolic phenotypes. One study detected higher levels of adiponectin and chemerin in girls with TS (30). Another study detected elevated interleukin (IL)-6 levels in patients with TS (31), while elevated CRP levels were found in another TS population (32); these findings suggest an ongoing chronic state of inflammation in these individuals. Further work is required to assess the mechanism by which adipokine or inflammatory cytokines alter metabolic function in TS patients.

### Autoimmunity in TS Patients

The risk of AD in patients with TS is approximately 2-fold higher than that in the female general population and 4-fold higher than that in male general population. Goldacre et al. analyzed 2,459 women with TS and discovered that Hashimoto's thyroiditis, diabetes mellitus, and coeliac disease were significantly more common than that in the general population (33). Antithyroid antibodies are common, whereas celiac, diabetic, and adrenal antibodies are rare. Excessive autoimmune antibodies may be caused by defects in the X chromosome. Genes on the X chromosome, including the major histocompatibility complex located in the long arm, have been shown to regulate immune responses and alter immune tolerance (34). Recent studies have shown that the pathogenesis of TS is associated with HLA haplotypes, genetic factors, single nucleotide polymorphisms, and cytotoxic T lymphocyte-associated protein-4. The increased prevalence of AD in patients with TS is also attributable to X-chromosome haploinsufficiency, maternal origin of X chromosome, overproduction of pro-inflammatory cytokines (IL-6), reduction in anti-inflammatory cytokines (IL-10, TGF-β), or hypogonadism (35).

The proportion of TS patients with anti-GAD65 was shown to be 4%, which is slightly higher than the 1.1% prevalence among adults in the general population. A cross-sectional study of 107 Danish TS patients by Mortensen et al. suggested that non-diabetic anti-GAD-65-positive TS patients may eventually develop diabetes (36). Thus, the increased risk of DM in TS patients may be attributable to autoimmune damage of β-cells. Hence, GAD-65 antibody testing is recommended for all TS patients with newly developed diabetes. Because the occurrence of diabetes in TS patients is related to the immune system, studies based on Treg have investigated the prevention of type 1 diabetes in patients with TS (35). It is expected to reduce immune-related islet β-cell damage in patients with TS via immunological regulation, thereby reducing the incidence of diabetes in patients with TS.

### Lifestyle of TS Patients

TS is likely to lead to various complications including lifestyle-related diseases such as diabetes mellitus, hypertension, and dyslipidemia. In a survey of 492 patients with TS (age ≥17 years), the prevalence of diabetes in patients aged ≥20 years was 6.3%. This survey also demonstrated a close association of lifestyle-related diseases with the severity of obesity rather than the karyotypes (11). As TS is associated with an increased risk of obesity; obesity may in turn further disrupt normal glucose metabolism, lead to development of insulin resistance, and eventually accelerate the development of T2DM. Over the past 40 years, the incidence of preobesity and obesity has increased dramatically across all age-groups worldwide (12). Therefore, we speculate that the increased prevalence of diabetes among TS patients may be associated with the incidence of preobesity and obesity. Sienkiewicz-Dianzenza et al. (37) indicated that the level of physical activity of girls with TS was not affected; however, further research suggests that the typical features of TS such as growth deficit and abnormal body proportion may contribute to physical weakness in these girls. We believe that patients with TS have unique lifestyle, such as reduced physical activity and sedentary lifestyle. Other common risk factors for T2DM, such as social environment and unhealthy dietary habits, are also risk factors for development of obesity and hyperglycemia. For TS women, especially those who are obese, it is important to follow the nutritional instructions and exercise therapy to control their body weight to prevent or alleviate diabetes.

## Effect of Growth Hormone (GH) or EP (Estrogen/Progestin) Therapy

Short stature is the dominant physical characteristic of TS, which has been linked to the *SHOX* gene (38). Current data suggests that the short stature is not due to a lack of GH (39); however, GH replacement therapy was shown to increase the height of these patients (19). Clinically, the use of GH therapy is now a standard means to achieve normal height of TS patients, typically starting at the age of 4–6 years.

GH can disrupt normal insulin signaling, and may potentially alter glucose metabolism. High level of GH can reduce skeletal muscle glucose uptake and increase hepatic glucose production, which aggravates insulin resistance (40).

Whether GH replacement therapy increases the risk of T2DM in TS patients is not clear. A recent retrospective study of children with TS who used GH replacement therapy for 7 years found no significant change in insulin sensitivity or β-cell secretory capacity (41). In a study of rhGH replacement therapy in 28 Chinese children, follow-up of children with TS and IGT did not reveal aggravation of deranged glucose metabolism (42). It is hypothesized that GH may have an insulin-like function, with the exception of increasing blood sugar. GH-induced IGT may indirectly promote insulin secretion and enhance arginine and glucose-stimulated insulin secretion, which may explain the lack of worsening of glucose metabolism during treatment. In another study (43), TS children treated with GH were found to have lower subcutaneous and visceral fat content and improved glucose tolerance than those who did not receive GH treatment; this suggests a protective effect of GH against metabolic dyshomeostasis in TS patients.

In another study, insulin sensitivity was slightly reduced in patients with TS at 4 years of initial GH therapy, especially during the first 6–12 months; the insulin sensitivity remained relatively stable in the subsequent 7–8 years of GH treatment, which may be related to an increase in lean body mass and a decrease in fat content (19). However, with the completion of GH treatment, insulin sensitivity is likely to recover slightly, although it cannot be restored to the pre-treatment level. Studies have shown that this increase in insulin sensitivity is not affected by BMI or triglyceride levels, but is related to the age at which GH therapy is discontinued. Although hyperinsulinemia and insulin resistance caused by GH therapy are reversible, the long-term effects are still unknown. Bannik et al. followed up TS patients after the completion of GH therapy (mean: 4.8 ± 1.9 years). Compared with pre-treatment, fasting insulin level was still high and insulin sensitivity was still low (44).

Another common characteristic of TS is hypergonadotropism, resulting in amenorrhea. As such, in order to induce puberty and development of female sex characteristics, EP (Estrogen /Progestin) therapy is typically required for TS patients. Estrogen replacement therapy in these individuals is initiated around the age of 12 years, with progesterone added to the therapeutic program within the first 2 years (2).

Seventeen-estradiol signaling is beneficial for energy homeostasis, skeletal muscle, adipose tissue, liver, pancreas, as well as the cardiovascular system (45). Animal research suggests that estrogen may suppress β-cell apoptosis (46) and may further regulate gene expression pertaining to insulin secretion and glucose uptake (47, 48). Human studies have further suggested that HRT may protect postmenopausal women against DM by counteracting the deficiency of insulin (49, 50).

Whether HRT may have some additional effect on glucose intolerance or development of DM in TS patients is not well-understood. Some studies have shown that estrogen replacement therapy can aggravate insulin resistance (7, 51). Gravholt et al. compared 24 women with TS treated with EP therapy and age-matched healthy women; they found that sex hormone replacement therapy increased IGT and the incidence of insulin resistance in the TS group. Giordano et al. conducted a study of adult women with TS (mean age: 32.4 ± 1.3 years) who received EP therapy and age-matched healthy women; they found that therapy increased coronary heart disease, IGT, insulin resistance, and hypertriglyceridemia in patients with TS (14). However, other similar studies found no impact of EP therapy on metabolic parameters (such as weight gain or glucose tolerance) in TS patients (52, 53). A study showed that 24-month estrogen replacement therapy has no significant effect on blood glucose, insulin sensitivity, or insulin levels in patients with TS; in addition, blood glucose levels may even be slightly reduced with increasing estrogen dose. The authors claimed that EP therapy may have a slight beneficial effect on glucose metabolism in patients with TS. In addition, the macrophage marker SCD 163, which is associated with insulin resistance and is elevated in TS patients, may reduce to some extent in patients receiving estrogen therapy (54). Thus, estrogen may alleviate the inflammatory state in TS patients and play a beneficial role in the maintenance of glucose homeostasis. Another study (55) did detect a trend of reduced arm fat mass (*p* = 0.054) and increase in total lean mass (*p* = 0.054) and trunk lean mass (*p* = 0.074) in patients utilizing estradiol gel. This may have been the consequence of the mode of percutaneous administration that bypasses the first liver metabolism, thereby allowing for smaller doses that are less likely to disrupt normal hepatic metabolic activity. However, guidelines established in 2017 (2) indicated that the superiority of transdermal (TD)estrogen to oral estrogen administration still remains controversial.

Given this uncertainty and the definitive efficacy of GH and EP treatment, annual monitoring for glucose tolerance based on glycosylated hemoglobin levels is recommended for all TS patients.

## Conclusion

**Figure 1:**
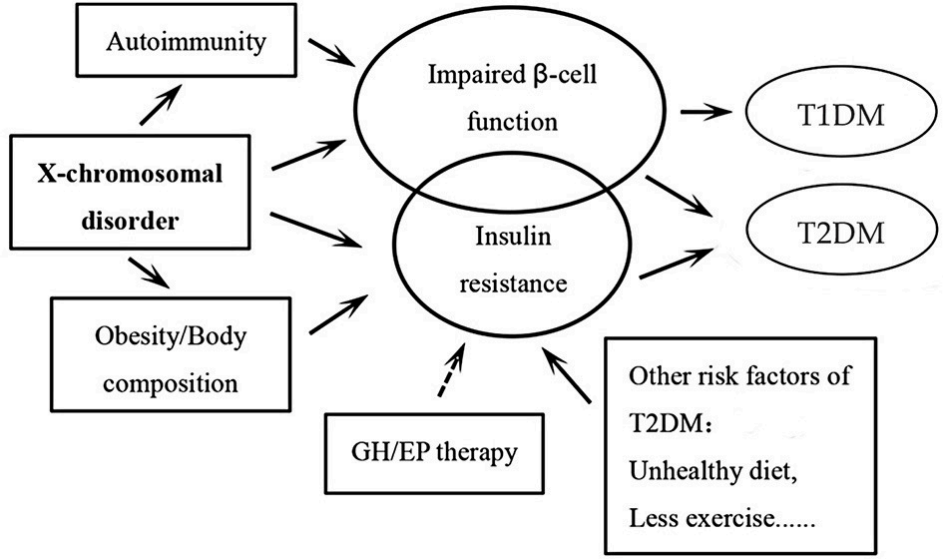
Schematic illustration of the pathogenetic mechanisms of the developmen*t of diabetes mellitus* (DM) in patients with Turner syndrome. The circle of impaired β-cell function is bigger than the one of insulin resistance, as we believe that insulin secretory response to glucose is a facet of TS that likely underlies the elevated risk of DM in these patients. It is the same for the circles of type 1 DM and type 2 DM. Among all the pathogenetic factors, X-chromosomal disorder may play the most important role. The arrows “→ ” indicate that more evidence may still be needed in this respect. GH: growth hormone; EP: Estradiol and progesterone; T1DM, type 1 diabetes mellitus; T2DM, type 2 diabetes mellitus.

Women affected by TS are at a significantly higher risk of DM. The underlying pathogenetic mechanism of DM development in TS is illustrated in Figure 1. Glucose intolerance appears to be an intrinsic defect associated with TS, although the genetic mechanisms are yet to be completely understood. Autoimmune factors may be involved in this process, although there is no definitive evidence in this respect. The traditional risk factors for type 2 DM in the general population (obesity, high visceral adipose content) do not seem to be a major factor in TS, especially in children. Growth hormone or estradiol/prosgesterone therapy in TS patients during childhood or adolescence may not alter the metabolic derangement or lead to development of DM in a destructive feedback cycle (1). Additional long-term prospective studies are required to verify these results.

In addition to disease-intrinsic DM risk factors, unhealthy lifestyle can also influence the development of DM. It is therefore important for TS patients to undergo regular weight monitoring, assessment of metabolic risk factors, and counseling pertaining to nutrition and physical activity. Further high quality RCT studies will provide additional evidence for the effective management of metabolic diseases in patients with TS.

## Author Contributions

GW devised the main concept of the manuscript. LS and XG collected data and wrote the first draft of the manuscript. YaW and TZ contributed to editing this work. XZ and YiW contributed to the revision of draft. All the authors have read and approved the submitted version.

### Conflict of Interest Statement

The authors declare that the research was conducted in the absence of any commercial or financial relationships that could be construed as a potential conflict of interest.
